# Optimized Directed Virus Evolution to Accelerate the Generation of Oncolytic Coxsackievirus B3 Adapted to Resistant Colorectal Cancer Cells

**DOI:** 10.3390/v16121958

**Published:** 2024-12-20

**Authors:** Leslie Elsner, Babette Dieringer, Anja Geisler, Maxim Girod, Sophie Van Linthout, Jens Kurreck, Henry Fechner

**Affiliations:** 1 Department of Applied Biochemistry, Institute of Biotechnology, Technische Universität Berlin, 13355 Berlin, Germany; l.elsner.1@tu-berlin.de (L.E.); babette.dieringer@tu-berlin.de (B.D.); a.geisler@tu-berlin.de (A.G.); maxim.girod@campus.tu-berlin.de (M.G.); jens.kurreck@tu-berlin.de (J.K.); 2 Berlin Institute of Health (BIH) at Charité-Universitätsmedizin Berlin, BIH Center for Regenerative Therapies (BCRT), 13353 Berlin, Germany; sophie.van-linthout@bih-charite.de; 3 German Center for Cardiovascular Research (DZHK), Partner Site Berlin, 13092 Berlin, Germany

**Keywords:** Coxsackievirus B3, PD-H, oncolytic virus, directed viral evolution, colorectal carcinoma

## Abstract

Recently, we demonstrated that the oncolytic Coxsackievirus B3 (CVB3) strain PD-H can be efficiently adapted to resistant colorectal cancer cells through dose-dependent passaging in colorectal cancer cells. However, the method is time-consuming, which limits its clinical applicability. Here, we investigated whether the manufacturing time of the adapted virus can be reduced by replacing the dose-based passaging with volume-based passaging. For this purpose, the murine colorectal carcinoma cell line MC38, resistant to PD-H-induced lysis, was initially infected with PD-H at 0.1 multiplicity of infection (MOI). For subsequent passages, 15–30 µL of a 1:10 dilution of the cell culture supernatant was transferred to fresh MC38 cells early after virus-induced cell lysis became visible. By virus passage 10, complete cell lysis of MC38 cells was achieved. Sequencing of the passage 10 virus (P-10) revealed two nucleotide substitutions in the 5′ UTR and six amino acid changes in the viral polyprotein compared to the PD-H founder. P-10, however, consisted of a heterogeneous virus population. Therefore, the detected mutations were introduced into the cDNA of PD-H, from which the recombinant virus PD-MC38 was generated. PD-MC38 exhibited significantly enhanced replication and lytic activity in MC38 cells compared to PD-H, whereas its oncolytic activity in other colorectal cancer cell lines was comparable to or even lower than that of PD-H. These findings demonstrate that volume-based passaging is suitable to generate tumor cell-specific adapted PD-H. Moreover, compared to the dose-dependent passaging, volume-based passaging significantly reduced the time required to generate the adapted virus.

## 1. Introduction

Oncolytic virotherapy is an innovative cancer treatment whose effectiveness has been demonstrated in numerous clinical trials over the past two decades. Key milestones include the approval of T-Vec, a modified herpes simplex virus (HSV), for melanoma treatment [1]; Oncorine, an modified adenovirus for the treatment of nasopharyngeal and head and neck cancers [2,3]; and Delytact, a HSV for the treatment of glioma [4]. The mechanism of oncolytic viruses (OVs) is based on the destruction of tumor cells through viral replication, accompanied by the stimulation of both the innate and adaptive immune responses, resulting in a systemic anti-tumor effect [5].

Despite the broad tropism exhibited by OVs, the effectiveness of treatment varies significantly, with only a small proportion of patients benefiting from OV monotherapy [6]. This variability stems at least in part from differences in cancer cell sensitivity, ranging from highly susceptible to completely resistant [7,8,9,10,11]. The underlying reasons for the resistance of tumor cells to OVs are diverse and often poorly understood. They include inefficient virus uptake due to a lack of or low expression of virus receptors on the surface of the target cells, lack of support for the viral replication machinery due to dysregulated cellular signal cascades, and insufficient release of the OV from the infected tumor cell due to inefficient induction of cell death [5,12].

To enhance the efficacy of OVs, genetic engineering and the incorporation of transgenes into the virus genome are frequently employed [13]. For instance, T-VEC has been engineered to express the transgene encoding granulocyte-macrophage colony-stimulating factor. This modification enhances anti-tumor immunity by promoting the recruitment and activation of dendritic cells, thereby improving antigen presentation and eliciting a more potent immune response against tumor cells [14]. Directed virus evolution (DVE) is an alternative strategy to overcome cancer cell resistance to OVs and enhance their efficacy. It includes the adaptation of an OV to the specific cancer cells by passaging the virus on the resistant cancer cells [15]. The duration of this process can vary, but it usually requires between 10 and 50 virus transfers [11,16,17,18,19,20,21]. This approach exploits the naturally high mutation rate of viruses during replication. Crucially, this method does not require extensive knowledge of virus–cell interactions or the specific factors that negatively impact viral replication. Instead, it exploits the inherent ability of the virus to adapt to the environment provided by the tumor cell in an autonomous microevolutionary process [15,22]. The resulting adapted viruses typically exhibit specificity for the cancer cells they were selected on [23] but can also show improved fitness across different cancer cells [17,18]. Thus, OVs generated through DVE appear particularly well-suited for use in personalized cancer medicine, especially for treating cancer types where the tumor cells exhibit primary resistance to OVs [23]. However, the practical implementation of the development of tumor cell-specific adapted OV in personalized medicine is complicated by the fact that its development takes a long time [17], which is often incompatible with the need for rapid availability of the OV in clinical practice.

Coxsackievirus B3 (CVB3) is a non-enveloped, positive-sense, single-stranded RNA virus of the genus *Enterovirus* within the family *Picornaviridae* [24]. Its oncolytic activity was first demonstrated in non-small cell lung carcinomas and subsequently in colorectal, breast, pancreas and cervical squamous cell carcinomas in vivo using various oncolytic CVB3 strains [8,25,26,27,28,29,30]. Certain oncolytic CVB3 strains have been shown to cause myocarditis and pancreatitis in vivo. However, this could be successfully mitigated by the incorporation of specific microRNA target sequences into the viral genome [31,32,33]. Recently published studies have shown that the efficacy of cancer therapy with oncolytic CVB3 can be enhanced by combining the oncolytic CVB3 treatment with other cancer therapies such as chemotherapy and immunotherapy [25,34]. The adaptation of the virus to resistant tumor cells, as recently performed by our group, is another effective approach to increase the anti-cancer efficacy of oncolytic CVB3 [23]. To this end, we have developed a protocol that enables the efficient and rapid generation of tumor cell-adapted oncolytic CVB3 from the CVB3 founder strain PD-H [23].

In this study, we investigated whether and to what extent the generation of tumor cell-specific adapted PD-H can be further accelerated. For this purpose, we replaced the dose-dependent passaging used in our previous protocol with volume-based passaging to adapt PD-H to resistant colorectal MC38 tumor cells. We demonstrate that the adapted virus PD-MC38, produced using this method, exhibited significantly higher replication and lytic activity in MC-38 cells compared to PD-H. Moreover, the time required for the generation of the tumor cell-adapted virus could be significantly reduced.

## 2. Materials and Methods

### 2.1. Cell Culture

HeLa cells were maintained in Eagle’s minimal essential medium (MEM) (Gibco, Karlsruhe, Germany) supplemented with 5% fetal calf serum (FCS), 0.02 M HEPES, 1% non-essential amino acids (NEAA), and 1% penicillin/streptomycin (P/S). HEK293T cells were grown in high-glucose DMEM (Biowest, Darmstadt, Germany) with 10% FCS, 1% P/S, 1% L-glutamine, and 1% sodium pyruvate. The murine colorectal carcinoma cell line Colon-26 and the human colorectal carcinoma cell lines Colo205, Colo320, and DLD-1 were cultured in RPMI 1640 (c.c.pro, Oberdorla, Germany) supplemented with 10% FCS, 1% P/S, 1% L-glutamine, and 2% sodium pyruvate. The human colorectal cancer cell line CaCo-2 was grown in high glucose DMEM (Biowest) supplemented with 10% FCS, 1% P/S, 1% L-glutamine, 1% NEAA, and 1% sodium pyruvate. The murine colorectal cancer cell line MC38 was cultured in high-glucose DMEM (Biowest) supplemented with 10% FCS, 1% P/S, 1% L-glutamine, 1% NEAA, 1% sodium pyruvate, and 0.01 M HEPES. The murine colorectal carcinoma cell line CT-26Luc was cultured in RPMI 1640 (c.c.pro) supplemented with 10% FCS, 1% P/S, 1% L-glutamine, 1% NEAA, and 2% sodium pyruvate.

### 2.2. Serial Passaging of PD-H in MC38 Cells

For serial passaging, 1.75 × 10^5^ MC38 cells per well were seeded in 24-well plates. Twenty-four hours later, the cells were infected with PD-H at an MOI of 0.1 for passage 1 (P1) or with 15 µL (P-2), 20 µL (P-7), 25 µL (P-9), or 30 µL (P-3, P-4, P-5, P-6, P-8, P-10) of a 1:10 dilution of virus-containing cell culture supernatant from the previous passage. Before adding the virus solution to the MC38 cells, the volume was adjusted to 100 µL with MC38 cell culture medium. The cells were incubated with the virus solution for 48 h (P-5, P-6, P-8, P-10) or 72 h (P-1, P-2, P-3, P-4, P-7, P-9) until cell lysis was visible. The viruses were isolated by three freeze–thaw cycles, and cell debris was removed by centrifugation. Viruses were stored at −20° until use.

### 2.3. Virus Plaque Assay

For the determination of virus titer, HeLa cells were seeded as a confluent monolayer in 24-well plates. After 24 h, the medium was removed, and the cells were incubated for 30 min with 300 µL of a 10-fold serial dilution of the virus samples in phosphate-buffered saline. The supernatant was removed, and cells were overlaid with 3.2% BD-Difco Noble Agar (Thermo Fisher Scientific, Waltham, MA, USA) containing MEM (Gibco, Karlsruhe, Germany). Plaque staining was carried out with Tetrazoliumbromid-Iodnitrotetrazoliumchlorid solution (both VWR International GmbH, Darmstadt, Germany) 72 h after virus infection.

### 2.4. Virus Growth Curves

Virus replication was examined via viral growth curves. For this purpose, 4 × 10^4^ MC38 cells were seeded in 96-well plates. Twenty-four hours later, the medium was removed and the cells infected with 100 μL virus suspension at MOI 0.1. After incubation for 1 h, the medium was removed and 150 µL fresh medium was added. At 0, 8, 24, 48, and 72 h post-infection, the cells were subjected to three freeze–thaw cycles. Cell debris was removed by centrifugation, and the supernatant was used to determine virus titers via plaque assay.

### 2.5. Cell Viability Assay

Cell viability was determined using the Cell Proliferation Kit (XTT) (Promega GmbH, Walldorf, Germany), following the manufacturer’s protocol. Briefly, 4 × 10^4^ MC38 cells were seeded in 96-well plates and infected the following day when they reached a confluence of about 70%. Absorbance was measured at 24, 48, and 72 h post-infection using the TriStar2 LB 942 Multimode Microplate Reader (Berthold Technologies, Bad Wildbad, Germany). As a control, cells were treated with 50 μL 5% Triton X-100 solution.

### 2.6. Mutagenesis and Cloning of PD-H cDNA-Containing Plasmids

To introduce the eight mutations (t249c, c736t, K112N, S348T, A661V, E768D, Y887C, I1477T) detected in the viral genome of P-10 into the cDNA of PD-H, the In-Fusion HD Cloning Kit (Takara Bio, Shiga, Japan) was used according to the manufacturer’s instructions. The Infusion Primer Design Tool (Takara Bio) was employed for the design of the mutagenesis primers. The plasmid pJet-CVB3-PD-H [24] served as the template for mutagenesis. The generated plasmid was termed pJET-CVB3-PD-MC38.

### 2.7. Generation of Recombinant PD-H and PD-MC38

For the generation of recombinant viruses, HEK293T cells were seeded into 6-well plates and transfected at a confluence of 70–80% with 2.5 μg of plasmids containing viral cDNA using PEImax (Polysciences Europe GmbH, Hirschberg an der Bergstraße, Germany). The virus PD-H was generated via the transfection of the plasmids pJET-CVB3-PD-H, and the virus PD-MC38 was generated via the transfection of the plasmid pJET-CVB3- PD-MC38. Cells were disrupted 72 h after transfection by three freeze–thaw cycles. Cell debris was removed, and the supernatant was used for determination of virus titers by plaque assay on HeLa cells.

### 2.8. Sequencing of CVB3 Genome

For the sequencing of the whole genome of P-10 and PD-H, viral RNA was extracted with a NucleoSpin RNA Virus Kit (Macherey-Nagel, Düren, Germany) according to the manufacturer’s instructions. Reverse transcription was carried out with the High-Capacity cDNA Reverse Transcription Kit (Applied Biosystems, Foster City, CA, USA). For the generation of PCR fragments, CloneAmp™ HiFi PCR Premix (Takara Bio) with CVB3-specific primers was used. Sanger Sequencing was conducted by LGC Biosearch Technologies (Berlin, Germany). SnapGene5.1.7 software (SnapGene, San Diego, CA, USA) was used for sequence alignment.

### 2.9. Comparison of PD-10 with Other CVB3 Strains

PD-H has been described in [26]. The sequences of additional CVB3 strains are accessible via the National Center for Biotechnology Information (NCBI) under the following accession numbers: strain 0 (AY752945.1), strain 20 (M88483.1), strain 2035 A (KY286529.1), strain 28 (AY752944.2), strain 31-1-93 (AF231763.1), strain H3 (U57056.1), strain L (M16572.1), strain M2 (M33854.1), strain Nancy (JX312064.1), strain P (AF231764.1), and strain RD (HQ157560.1).

### 2.10. Statistical Analysis

Statistical analysis was performed with Graph-Pad Prism 8.2 Software (GraphPad Software, Boston, MA, USA). The two-tailed unpaired Student’s *t*-test was used to determine statistical significance. Results are expressed as the mean ±SEM for each group. Differences were considered significant at *p* < 0.05.

## 3. Results

### 3.1. Adaptation of PD-H to MC38 Cells

**Figure 1:**
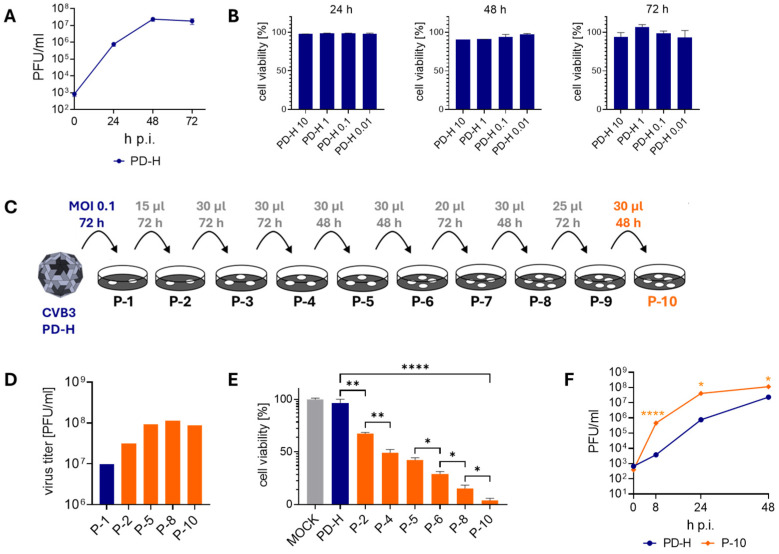
Adaptation of PD-H to MC38 cells. (**A**) Virus growth kinetics. MC38 cells were infected with PD-H at MOI 0.1. Virus titer was determined by plaque assay 24 h, 48 h, and 72 h p.i. Shown are mean values ± SEM (n = 3). (**B**) Cell viability of MC38 cells after infection with PD-H. MC38 cells were infected with PD-H at the indicated MOI. Cell viability was measured by XTT assay after 24 h, 48 h, and 72 h. Cell viability was normalized to untreated cells (=100%). Shown are mean values ± SEM (n = 3). (**C**) Scheme of the serial passaging of PD-H on MC38 cells. For P1, cells were infected with PD-H at MOI 0.1 and incubated for 72 h. For further passaging to P10, the stated amount of a 1:10 dilution was added to fresh MC38 cells and incubated for the indicated time. (**D**) Development of viral titers during serial passaging of PD-H on MC38 cells. The virus titers were determined by plaque assay on HeLa cells. (**E**) Changes in the ability of PD-H to lyse MC38 cells during passaging. MC38 cells were infected with PD-H, P-2, P-4, P-5, P-6, P-8, and P-10 at MOI 1. Cell viability was measured by XTT assay 48 h after infection. Shown are mean values ± SEM (n = 3). Significance, * *p* < 0.05; ** *p* < 0.01; **** *p* < 0.0001. (**F**) Virus growth kinetics. MC38 cells were infected with PD-H and P-10 at MOI 0.1. Virus titer was determined by plaque assay. Shown are mean values ± SEM (n = 3). Significance compared to PD-H, * *p* < 0.05; **** *p* < 0.0001.

To assess the susceptibility of MC38 cells to PD-H, we first examined the replication and oncolytic activity of the virus in these cells. PD-H replicated strongly in MC38 cells (Figure 1A) but had low oncolytic activity (Figure 1B). Even 72 h post-infection (p.i.) with a high MOI of 10, only a slight decrease in cell viability was observed. To improve the performance of PD-H in MC38 cells, we adapted the virus to the cells by serial passaging (Figure 1C). For this purpose, a defined volume of cell culture supernatant of infected MC38 cells was transferred to new MC38 cells. This approach eliminates the time-consuming repeated virus titration often used in protocols to adapt viruses to resistant cells [17,23]. The initial infection of the cells was conducted with an MOI of 0.1 for 72 h. For all further infections, a volume between 15 and 30 µL of a 1:10 dilution of the previous passage was used. The virus-containing cell supernatant was collected early after cell lysis became visible, which typically occurred 48 or 72 h after the cells were infected (Figure 1C). Serial passage of PD-H into MC38 cells led to a continuous increase in viral titer, reaching a level approximately 10-fold higher than that of the PD-H founder. The maximum increase in virus titer was observed after passage 5, with no further increase observed up to passage 10, at which point passaging was terminated (Figure 1D). To assess how passaging influenced the oncolytic potential of PD-H, an XTT assay was performed, revealing a significant enhancement in cytolytic activity. This led to the complete lysis of MC38 cells after infection with the P-10 using an MOI of 1 and a 48 h incubation period (Figure 1E). The viral growth kinetics of the P-10 virus revealed a significant 10- to 100-fold increase in replication compared to the PD-H strain in MC38 cells (Figure 1F). Thus, the volume-based passaging approach facilitated the successful adaptation of PD-H to MC38 cells. Compared to our previous approach [23], the time required to generate tumor cell-adapted P-10 virus was reduced by more than 50%, from more than 60 days to less than 30 days.

### 3.2. Sequence Analysis of P-10 and Comparison of P-10 with Other CVB3 Isolates

**Figure 2:**
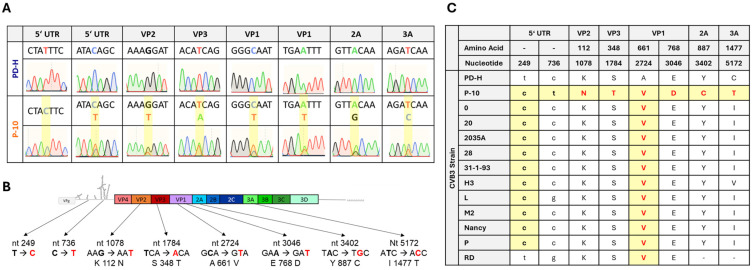
Sequence analysis of P-10 and comparison of P-10 with other CVB3 isolates. (**A**) Analysis of mutations that occurred in P-10. Shown are eight electropherograms of the sanger sequencing of viral RNA isolated from PD-H and P-10. Nucleotide substitutions are highlighted in yellow boxes. For seven mutations, a double signal in sanger sequencing was detected. (**B**) Scheme of the genome of PD-H. The location of the mutations, the mutated nucleotides, and the resulting amino acid substitutions detected in P-10 are indicated. (**C**) Comparison of the nucleotide and amino acid substitutions found in PD-10 with other CVB3 strains. Only a partial cDNA is available for the RD strain; no comparison was carried out for the mutations in 2A and 3A protein.

To determine which mutations lead to the adapted phenotype of P-10, the genomic RNA of P-10 was isolated, reverse transcribed, and sequenced. Eight nucleotide substitutions were detected (Figure 2A,B). Two of them were found in the 5′ UTR and a further six, leading to amino acid substitutions, were detected in the viral polyprotein. Four of them occurred in the viral capsid proteins VP1, VP2, and VP3 and two of them in the non-structural proteins 2A and 3A (Figure 2B). Sequencing revealed double signals in the electropherogram for seven out of the eight detected mutations, indicating that, in addition to the mutated PD-H variant, at least one other virus with a genome sequence closely resembling that of PD-H was present in P-10. Thus, P-10 consisted of a mixed virus population. To further investigate the significance of the detected mutations, we compared the mutations in P-10 with the corresponding nucleotide or amino acid sequences in known CVB3 isolates. The analysis revealed that the mutation t249c in the 5′ UTR and the mutation A661V in the VP1 protein were present in the majority of other CVB3 isolates, whereas the remaining mutations were specific to P-10 (Figure 2C). This indicates that the adaptation of PD-H to MC38 has produced a unique CVB3.

### 3.3. Replication and Oncolytic Activity of PD-MC38

**Figure 3:**
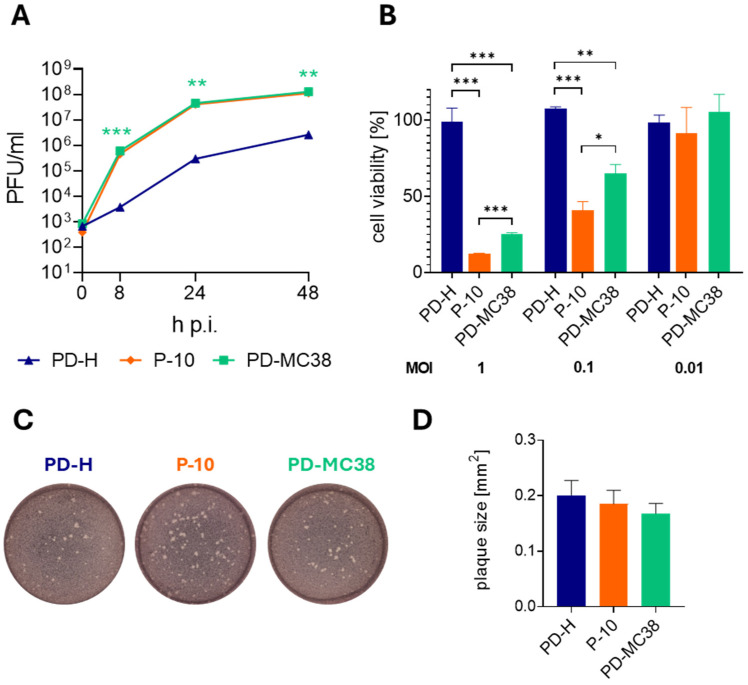
Replication and oncolytic activity of PD-MC38. (**A**) Virus growth kinetics. MC38 cells were infected with PD-H, P-10, and PD-MC38 at MOI 0.1. Virus titer was determined by plaque assay. Shown are mean values ± SEM (n = 3). There was no significant difference between P-10 and PD-MC38. Significance of PD-MC38 compared to PD-H, ** *p* < 0.01; *** *p* < 0.001. (**B**) Cell viability of MC38 cells after infection. MC38 cells were infected with PD-H, P-10, and PD-MC38 at the indicated MOI. Cell viability was measured by XTT assay 48 h later. Cell viability was normalized to untreated cells (=100%). Shown are mean values ± SEM (n = 3). Significance, * *p* < 0.05; ** *p* < 0.01; *** *p* < 0.001. (**C**) Representative images of viral plaques (white dots) of PD-H, P-10, and PD-MC38 on HeLa cell monolayers. (**D**) Graphical representation of plaque size of PD-H, P-10, and PD-MC38 shown under C. Shown are mean values ± SEM for n = 50 plaques.

Based on our previous observation that mutations acquired during the adaptation of PD-H to tumor cells enhance viral activity when acting together [23], all mutations identified in P-10 were introduced into the genome of the PD-H strain. The virus PD-MC38, containing these mutations in its genome, was produced through transfection of the respective viral cDNA into HEK293T cells. To access the oncolytic properties of PD-MC38, we next compared its replication and oncolytic activity with that of PD-H and P-10 in MC38 cells. Following infection at MOI 0.1, PD-MC38 showed similar replication to P-10 but significantly stronger replication than PD-H, indicating that the insertion of the mutations was beneficial for viral replication (Figure 3A). To investigate the oncolytic activity of all three viruses, we infected MC38 cells with PD-MC38, PD-H, and P-10 at an MOI of 1, 0.1, and 0.01 and measured cell viability 48 h later by XTT assay. At a very low dose of 0.01 MOI, the three viruses did not show oncolytic activity. However, at higher doses of 0.1 and 1 MOI, treatment with PD-MC38 induced a pronounced decrease in MC38 cell viability, reducing it to 65% and 25%, respectively, whereas PD-H had no effect on cell viability. Nevertheless, PD-MC38 did not achieve the same level of activity as P-10, which reduced cell viability to 40% at 0.1 MOI and to 12% at 1 MOI (Figure 3B). A comparison of plaque morphology and plaque size between PD-H, P-10, and PD-MC38 showed no significant differences (Figure 3C,D), indicating that the acquired mutations did not affect these parameters.

### 3.4. Oncolytic Efficiency of PD-MC38 in Different Colorectal Cancer Cell Lines

**Figure 4:**
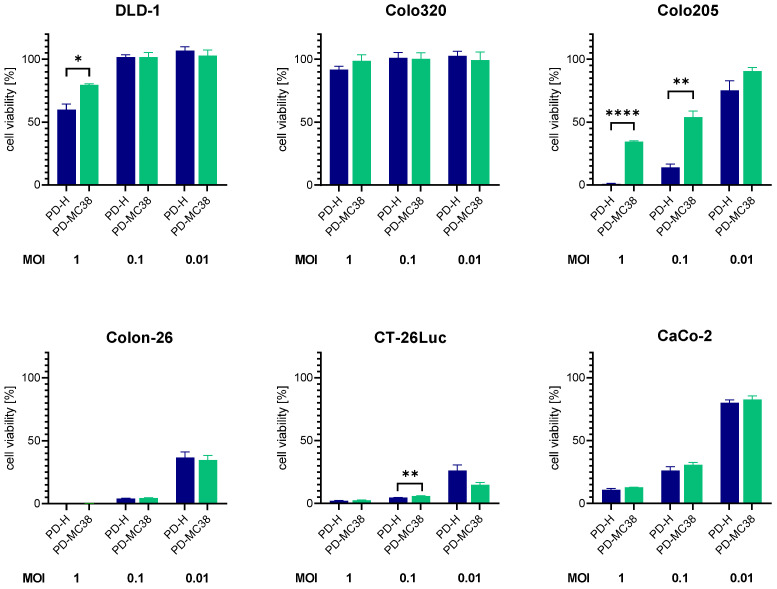
Comparison of oncolytic efficiency of PD-MC38 and PD-H in different colorectal cancer cell lines. The colorectal cancer cell lines DLD-1, Colo320, Colo205, CaCo-2, Colon-26 and CT-26Luc were infected with PD-H and PD-MC38 at the indicated MOI; cell viability was measured 48 h after infection with XTT assay. Shown are mean values ± SEM (n = 3). Significance, * *p* < 0.05; ** *p* < 0.01; **** *p* < 0.0001.

To verify whether the adaptation is specific only for MC38 cells, we infected six additional colorectal carcinoma cell lines with PD-M38 and PD-H at 0.01, 0.1, and 1 MOI and determined the cell viability 48 h later by XTT assay (Figure 4). In four of the cell lines (Colo320, CT-26Luc, Colon-26, and CaCo-2), PD-MC38 showed similar cytotoxicity to PD-H, whereas in DLD-1 and Colo205 cells, the cytotoxicity of PD-MC38 was significantly lower than that of PD-H. These data demonstrate that the enhanced oncolytic activity of PD-MC38 is specific to MC38 cells.

## 4. Discussion

Personalized cancer therapy represents a transformative approach in oncology, moving away from traditional one-size-fits-all solutions towards strategies that are tailored to the individual characteristics of each patient’s cancer [35,36]. This consideration is based on the fact that tumors are phenotypically and genotypically very heterogeneous, and therefore, individualized therapeutic approaches promise the best therapeutic success. This assessment also applies to cancer therapy with OVs. However, personalized virotherapy strategies are still largely undeveloped, although preclinical studies have often shown that tumor cells have different sensitivities and individual resistances to existing OVs [10,37]. One of the reasons for this is the complexity of virus–tumor cell interactions, which makes it difficult to predict the efficacy of an OV based on selected tumor biomarkers.

**Figure 5:**
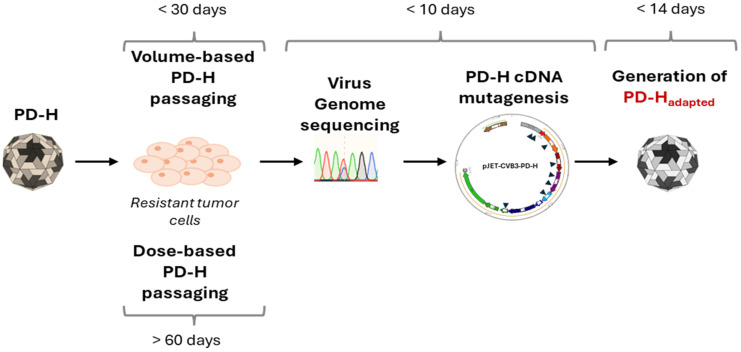
Schematic procedure and time schedule of the generation of tumor cell-specific adapted oncolytic CVB3 strain PD-H.

An alternative approach for the personalized application of OVs is the development of tumor cell-adapted OVs that selectively and efficiently target a patient’s tumors. In this context, several studies have demonstrated that OVs can be adapted to resistant tumor cells through repetitive virus passaging, enabling them to effectively target tumor cells [15,16,38,39]. However, the protocols for developing the adapted OV vary widely, and the overall development process suffers from the fact that it generally takes a long time to produce the customized OV [15,17]. The latter is a decisive disadvantage of this technology and crucial for its employment, as it is questionable whether a cancer patient could still benefit at all from the development of such an OV. Addressing this problem, we recently established a protocol enabling rapid generation of adapted oncolytic CVB3 PD-H [23]. This was achieved by limiting the virus passaging to 10 cycles and by inserting all detected virus mutations directly into the cDNA of the founder virus PD-H. Here, we demonstrate that the time required for the generation of tumor cell-adapted PD-H can be further reduced. For this purpose, we replaced the dose-based passaging used in the protocol with a volume-based virus passaging procedure. The decisive advantage of this approach is that the time-consuming virus titration, which is necessary after each passaging cycle to determine the virus dose for the next infection, is replaced by the transfer of a defined volume of cell culture supernatant. Using this method, we show that PD-H can successfully be adapted to the murine colorectal cancer cell line MC38. Moreover, compared to the dose-based virus passaging, the volume-based virus passaging procedure reduced the time required for the generation of the adapted PD-H from more than 60 days to less than 30 days. Considering the total development time, which also includes genetic engineering of the founder PD-H and the final virus production, we assume that a tumor cell-adapted oncolytic PD-H variant can be produced in less than 2 months (Figure 5). However, it appears feasible to reduce the incubation time of the cell culture in each passage, which was 48 or 72 h in our study, to 24 h or even less, potentially accelerating the production of tumor cell-adapted oncolytic PD-H.

Another critical step in shortening the development time of adapted OVs is to minimize the number of viral passages. Here, the number of virus passages was limited to 10. Genome sequencing of the resulting virus P-10 identified several mutations. The majority of these mutations exhibited double signals, indicating a heterogeneous viral population comprising both the mutated variant and the founder strain PD-H. In such a case, further virus passaging or isolation of the mutated variant by plaque purification is usually carried out until the mutated variant is present in pure form [11,17,40]. We have shortened this process by inserting the detected mutations directly into the cDNA of PD-H. Upon generating the virus PD-MC38 via the transfection of HEK293T cells, the replication and lytic activity of PD-MC38 in MC38 cells were significantly enhanced compared to PD-H cells, confirming the successful generation of an adapted virus with our method. However, the cytotoxicity of PD-MC38 was somewhat lower than that of P-10, although the replication was similar. This presents some contrast to our previous study, in which the recombinant virus PD-SK, which was generated from P-10 after the adaptation of PD-H to the colorectal tumor cell line Colo320, showed even stronger replication and cytotoxicity than P-10 [23]. The reason for the difference in performance between the PD-SK and PD-MC38 viruses is not clear. However, it can be ruled out that the presence of a mixed viral population in P-10 in the presented study plays a crucial role, as such a population was also present in P-10 in our previous study.

We did not further investigate the impact of each mutation on the replication and cytotoxicity of PD-MC38, as this was not the primary focus of our study. Nevertheless, four of the eight mutations identified in the viral genome were located within the viral capsid proteins VP1, VP2, and VP3, suggesting that the adaptation of PD-H to MC-38 cells is likely associated with virus uptake into the cancer cells. This is supported by the observation that the Alanine → Valine amino acid substitution at position 661 of the viral polyprotein (amino acid 91 in VP1) was present in the majority of known CVB3 strains. It highlights the significance of Valine at this position for the function of VP1 in CVB3. Moreover, footprint analysis demonstrated that this amino acid is involved in the interaction of the viral capsid with the coxsackie and adenovirus receptor (CAR), which mediates the attachment and binding of CVB3 to target cells [41,42]. In PD-H strains, this interaction is weak [43]. Thus, it may be possible that the substitution of Alanine by Valin enhanced the binding of the adapted virus to CAR. The mutation t249c in the 5′ UTR could be similarly significant, as it has been found in the majority of CVB3 strains, but so far, it is uncharacterized regarding its function. Furthermore, the viral proteins 2A and 3A, which are involved in apoptosis induction and cellular replication, respectively [44,45], each acquired a single amino acid substitution during the adaption. The occurrence of the mutations in both proteins was closely correlated with our observation that the replication and lytic infection of P-10 and PD-MC38 were significantly enhanced in MC38 compared to PD-H, highlighting the importance of these mutations in improving the viral performance of the adapted virus.

Other colorectal cell lines investigated in this study showed equal or even worse susceptibility to PD-MC38 compared to PD-H, demonstrating that the adaptation of PD-H to MC38 cells is strictly tumor cell specific. A similar observation was made in an earlier study we conducted, in which PD-H was adapted to the human colon cancer cell line Colo320 [23]. This confirms the personalized character of the adaptation of PD-H to colorectal cancer cells.

In conclusion, we demonstrate that the adaptation of PD-H to a resistant colorectal tumor cell line can be expedited through the application of a volume-based passaging procedure. By incorporating the acquired mutations into the PD-H founder, a viral clone that fixes the characteristics of the adapted virus can be efficiently and rapidly generated. This method may contribute to enhancing the feasibility of developing PD-H for personalized therapies in patients. Moreover, given its potential applicability to other oncolytic RNA viruses, the utility of this approach could extend well beyond the oncolytic CVB3 PD-H model investigated in this study.

## Data Availability

Data will be supplied following reasonable requests.
